# More V.A.D.s of All Ranks Wanted

**Published:** 1916-08-05

**Authors:** Arthur Stanley

**Affiliations:** Chairman, Executive Committee British Red Cross Society.; Director of the Ambulance Department of the Order of St. John.


*To the Editor of* The Hospital.

### More V.A.D.s of all Ranks Wanted.

*To the Editor of* The Hospital.

Sir,—A real and urgent necessity has arisen for more nurses, V.A.D. nursing members (women), and V.A.D. general service members in military and auxiliary hospitals at home. The demands made upon us by the military authorities are very heavy, and cannot be met out of the existing supply. There must still be many women who are not giving the whole of their time and service to the war, and who have no ties which prevent them from doing so. We earnestly call upon these women to come forward and help us in this emergency, and thus enable us to answer the call of the sick and wounded men.

Suitable women who are willing to help in the hospitals may be attached to existing Voluntary Aid Detachments for immediate service in the hospitals.

Full information on this point may be obtained from the Women's Joint V.A.D. Committee, Devonshire House, or from the County Directors, Col. Valentine Matthews, Duke of York's Headquarters, Chelsea, or from Col. T. E. L. Bate, Craig's Court House, Whitehall.—Yours faithfully,

Arthur Stanley,
Chairman, Executive Committee British Red Cross Society.

Ranfurly,
Director of the Ambulance Department of the Order of St. John.

July 27, 1916.

